# Supplementary material for: Grave-to-cradle upcycling of Ni from electroplating wastewater to photothermal CO2 catalysis
Source: Nat Commun. 2022 Sep 9;13:5305. doi: 10.1038/s41467-022-33029-x (PMC9463155; doi:10.1038/s41467-022-33029-x)
Supplement: Supplementary file 1 — Supplementary Information [file 41467_2022_33029_MOESM1_ESM.pdf]

Supplementary Information

Grave-to-Cradle Upcycling of Ni from Electroplating Wastewater to Photothermal CO_2 Catalysis

Shenghua Wang[1†], Dake Zhang[1,2†], Wu Wang[3], Jun Zhong[2], Kai Feng[4], Zhiyi Wu[2], Boyu Du[5], Jiaqing He[3], Zhengwen Li[4], Le He[2,6✉], Wei Sun[1✉], Deren Yang[1✉], and Geoffrey A. Ozin[7✉]

Supplementary notes:

Synthesis of CNC@mSiO_2

100 mg of the CNC powder was dispersed into a mixture of 2.5 mL of Milli-Q water and 20 ml of the prepared CTAB solution (0.9 g of CTAB dissolved in 300 mL of Milli-Q water and 100 mL of ethanol). The suspension was sonicated and stirred, each for 20 min. 300 μL of ammonium hydroxide (28%) aqueous solution and 400 μL of TEOS were added into the suspension sequentially. The mixture was stirred for another 16.25 h in a shaking bed (400 rpm, 30 °C). The obtained nanoparticles were then separated and redispersed in acetone and refluxed at 80 °C for 48 h. The refluxing procedure was repeated 3 times. The obtained products (denoted as CNC@ mSiO_2) were then washed with ethanol several times and dried in a vacuum oven.

Synthesis of solid SiO_2 supported Ni nanoparticles (Ni_{im}@SiO_2)

The synthesis of Ni_{im}@SiO_2 was via a traditional impregnation method. 33 mg of $Ni(NO_3)_2$•$6H_2O$ was dissolved into 5 mL of ethanol, followed by the addition of 60

mg of solid silica nanospheres. The mixture was stirred under 80 °C to evaporate the solvent. The dried powders were further dried under vacuum and then calcined in air (500 °C, 1h) and H_2 (600 °C, 2h) sequentially.

Acid etching experiments

50 mg of a certain sample (CNC@SiO_2@mSiO_2 or CNC@mSiO_2) was dispersed into 5 mL of HCl solution (1 or 3 M). The suspension was transferred to a shaking bed (30 °C, 400 rpm) and sampled after 1 h, 3 h, 6 h, 12 h and 24 h, respectively.

The elimination of internal diffusion

The elimination of internal diffusion was verified by the Weisz-Prater criterion (**Equation S1**), where r represents the reaction rate per volume of catalyst (mol s^{-1} cm^{-3}), R represents the catalyst particle radius (cm), C_s represents the reactant concentration at the particle surface (mol cm^{-3}), and D_{eff} represents the effective diffusivity.[1,2]

The key to calculate N_{W-P} is to obtain the value of D_{eff} first, which can be predicted from Fuller-Schettler-Giddings method for binary gas phase diffusion. where D_{AB} is the binary gas phase diffusion coefficient (cm^2 s^{-1}), ε is the catalyst porosity, τ is the tortuosity factor, T is the reaction temperature (K), M_A and M_B is the molecular weight for gas A and B, P is the pressure (bar), ΣV_A and ΣV_B is the sum of diffusion volume for component A and B.[1,3]

$$N_{W-P} = \frac{rR^2}{C_s D_{eff}} \ll 0.3 \qquad \text{(Equation S1)}$$

$$D_{eff} = D_{AB}\varepsilon/\tau \qquad \text{(Equation S2)}$$

$$D_{AB} = 10^{-3}T^{1.75}(\frac{1}{M_A} + \frac{1}{M_B})^{\frac{1}{2}}/P[(\sum V_A)^{\frac{1}{3}} + (\sum V_B)^{\frac{1}{3}}]^2 \qquad \text{(Equation S3)}$$

In our case, A represents CO_2, B represents H_2. $T = 773$ K; $M_A = 44$; $M_B = 2$; $P = 1$ bar; $\Sigma V_A = 26.9$;[3] $\Sigma V_B = 7.07$;[3] The value of D_{AB} was calculated to be 3.3908.

$\tau = 4.5$;[1] $\varepsilon = 0.18$ (obtained from the BET results); The value of D_{eff} was calculated to be 0.1356.

Taking the reverse water-gas shift reaction (0.03 g of $S_{Fe\text{-}Ni}$, 773 K, CO_2: H_2 = 10: 30 mL/min) in our case as a typical example. The volume of the packed $S_{Fe\text{-}Ni}$ catalyst (0.03 g) was estimated to be ~0.07 cm^3, and the CO rate under this condition was determined to be ~0.017 mol g^{-1} h^{-1} (the selectivity towards CO was 99%). Therefore, $r \approx [0.017/(0.07/0.03)]/3600 \approx 2.02*10^{-6}$;

The catalyst powder was ≤ 80 mesh, therefore, $R_{max} \approx 0.018$;

C_s of CO_2 = (pV)/(RT) =1/4*(101325*10^{-6})/(8.314*773) ≈ 3.94*10^{-6};

The value of $N_{W\text{-}P}$ was calculated to be ~0.0012 < 0.3.

Therefore, the influence of internal diffusion was successfully eliminated.

The detailed calculations of the CO_2 footprint

The net CO_2 emission rate of thermocatalytic reaction was calculated based on the equation: $dM2_{\mathrm{kp}}/dt = (0.00048T\text{-}0.016)*11.04 - mc$. The net CO_2 emission rate (denoted as dM/dt, unit: mol h^{-1}) of photothermal catalytic reaction (light source: Xe arc lamp) was calculated based the equation: $dM1_{\mathrm{kp}}/dt = 11.04a - mc$. The net CO_2 emission rate of photothermal catalytic reaction (light source: sunlight) was calculated based the equation: $dM1_{\mathrm{kp}}/dt\text{-S} = -mc$. In the equations above, 'S' in '$dM1_{\mathrm{kp}}/dt$-S' represents that the light source is sunshine, a represents the power of the lamp (unit: kW), m represents the mass of the catalyst (unit: g), c represents the conversion rate of

CO_2 (unit: $mol \cdot g^{-1} \cdot h^{-1}$), and T represents the reaction temperature. All the equations were based on the assumption that the generation of H_2 is CO_2-free.

The calculations were based on our previous work. The CO rate of ~5 $mmol \cdot g^{-1} \cdot h^{-1}$ was found for $S_{Fe\text{-}Ni}$ at either a thermocatalytic process (500 °C) or a photothermal catalytic (190 W illumination assisted with a concentrator) process. Therefore, the values of a, m, c, and T for $S_{Fe\text{-}Ni}$ were set to be 0.19, 0.03, 0.005, and 500, respectively.

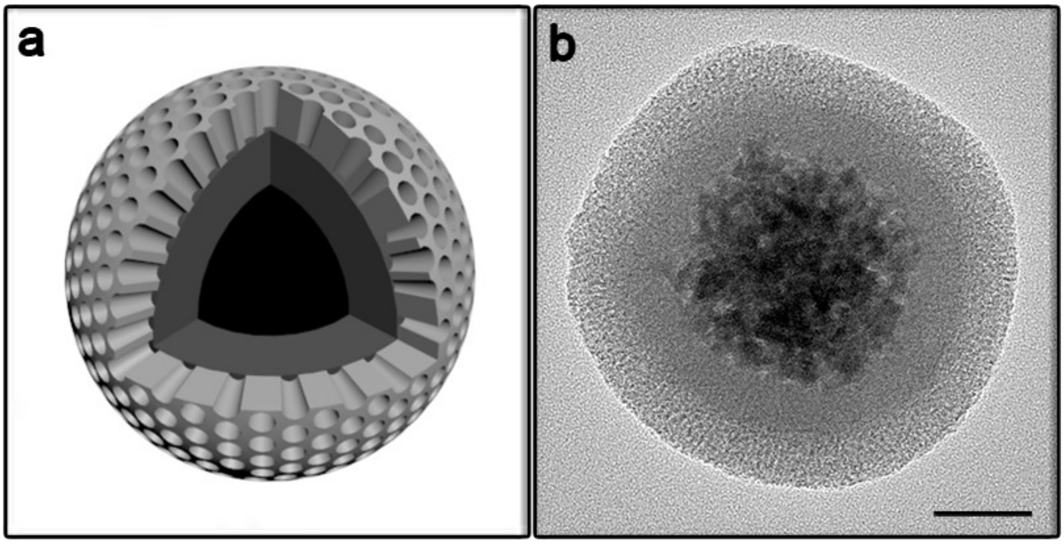

Supplementary Fig. 1 Morphology and components of S_{ad}. (a) Schematic illustration of the architecture of the S_{ad}. The black core, the dark grey middle layer, and the light grey mesoporous outermost layer represent the CNC, the dense silica, and the mesoporous silica, respectively. **(b)** TEM image of S_{ad}. Scale bar, 50 nm.

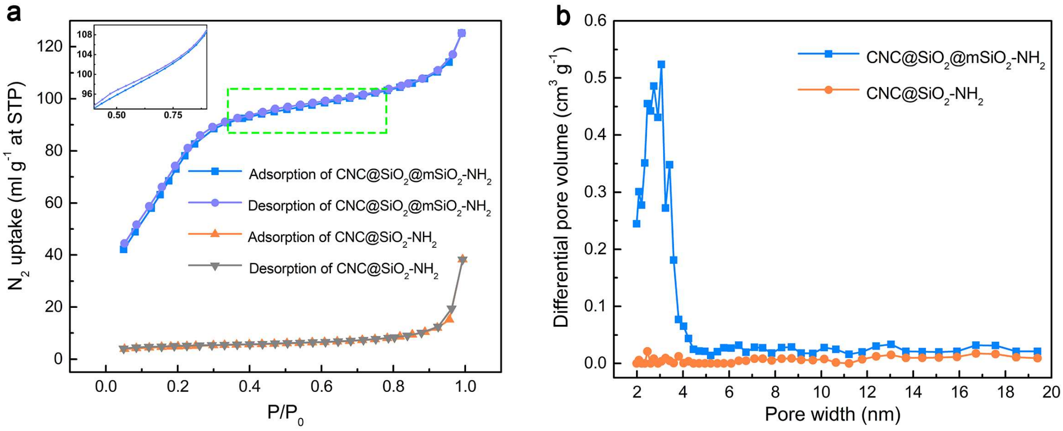

Supplementary Fig. 2 Enhanced specific surface area. (a) N_2 adsorption–desorption isotherms and **(b)** the corresponding pore size distributions of the amino-grafted adsorbents with and without the mesoporous silica layer (denoted as CNC@SiO_2@mSiO_2-NH_2 and CNC@SiO_2-NH_2, respectively). The insert in (a) is the enlarged graph of the enclosed area.

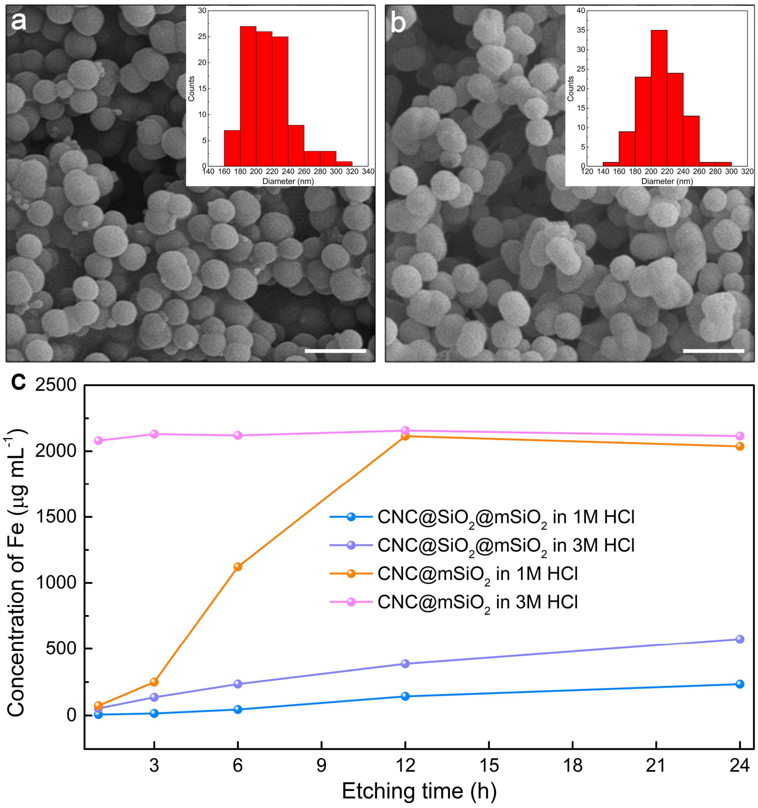

Supplementary Fig. 3 Enhanced acid resistance property. (a) Scanning electron microscope (SEM) image of CNC coated by a dense silica layer, followed by the coating of a mesoporous SiO_2 layer (denoted as CNC@SiO_2@mSiO_2). Scale bar, 500 nm. **(b)** SEM image of CNC coated by a mesoporous SiO_2 layer (denoted as CNC@mSiO_2). Scale bar, 500 nm. **(c)** The concentration of Fe in the etching solution. The inserts in (a) and (b) are the particle size distributions of the corresponding samples.

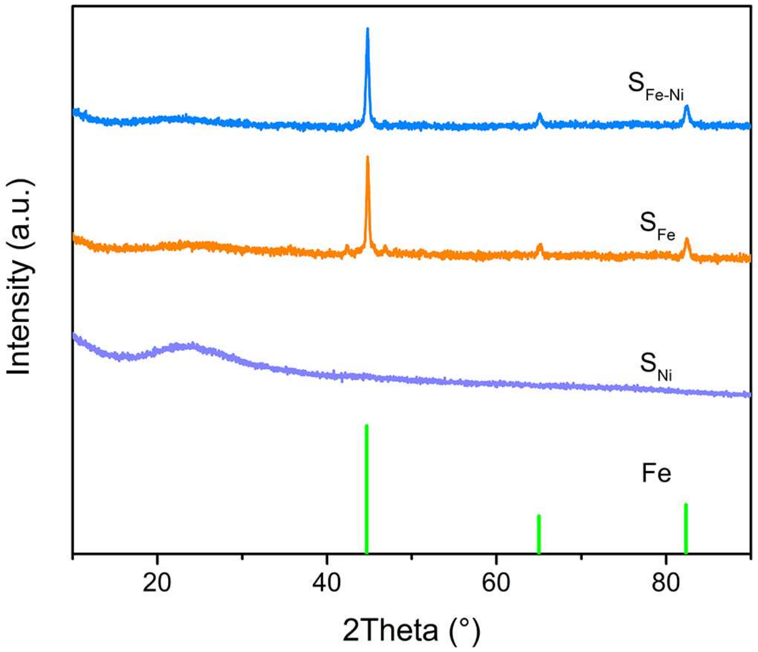

Supplementary Fig. 4 Evidence of the small size of Ni. XRD patterns of the different samples and the standard Fe (JCPDS 06-0696).

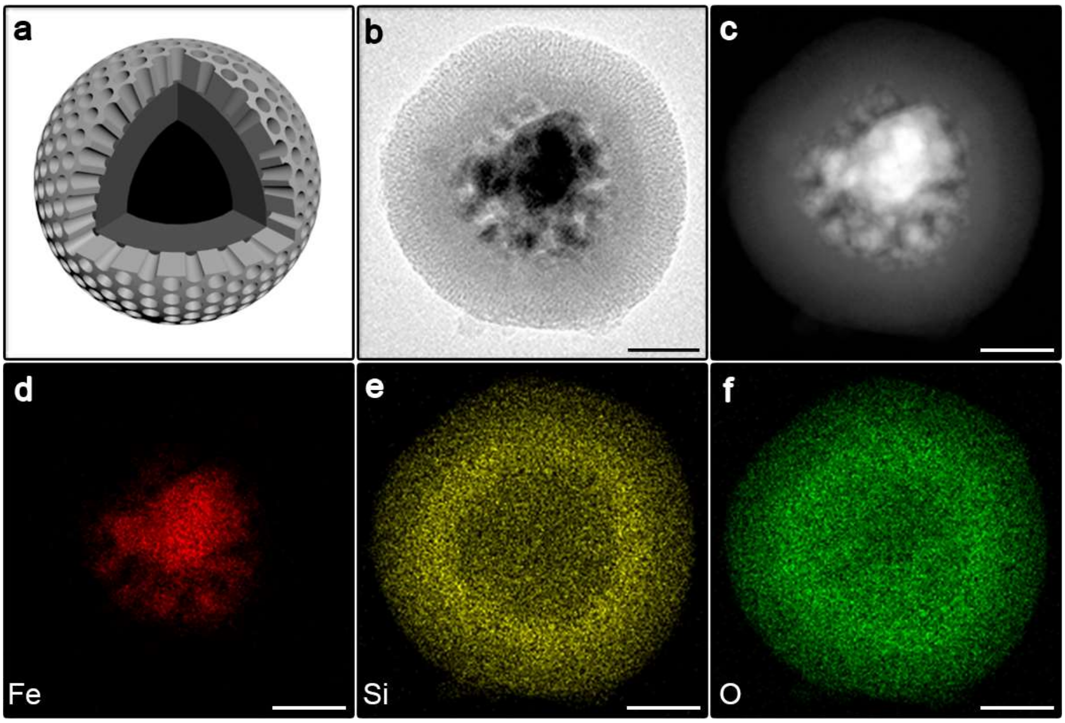

Supplementary Fig. 5 Morphology and components of S_{Fe}. (a) Schematic illustration of the architecture of S_{Fe}. The black core, the dark grey middle layer, and the light grey mesoporous outermost layer represent the CNC, the dense silica, and the mesoporous silica, respectively. **(b)** TEM image of S_{Fe}. Scale bar, 50 nm. **(c)** HAADF-STEM image of S_{Fe}. Scale bar, 50 nm. **(d–f)** EDS mappings of S_{Fe}. Scale bars, 50 nm.

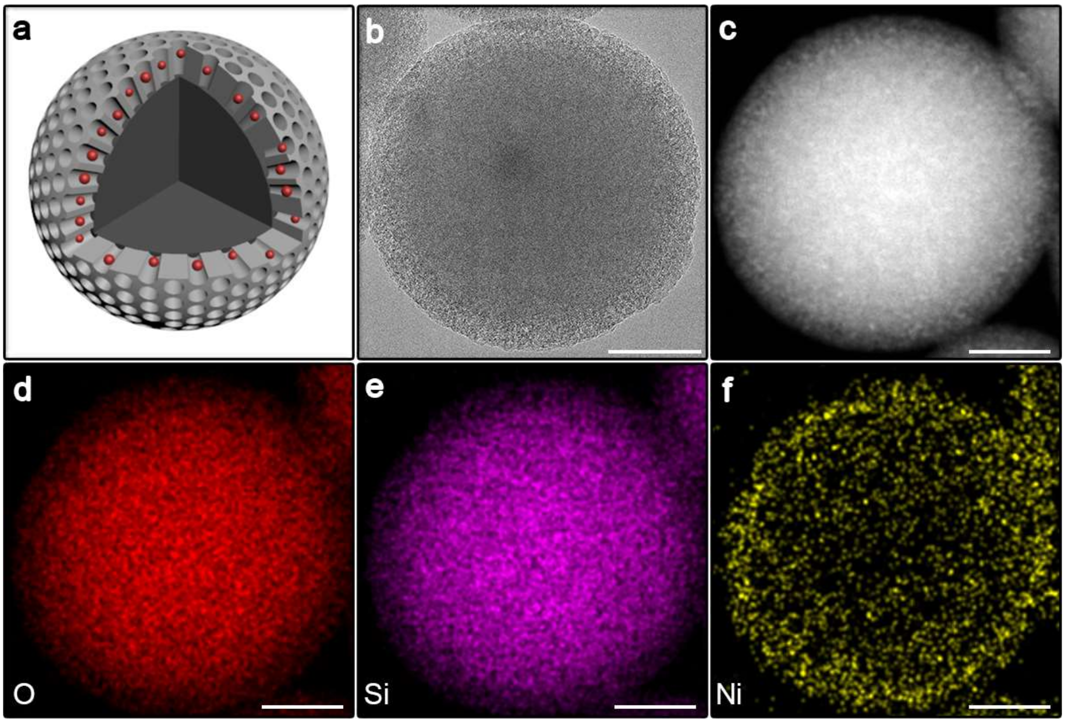

Supplementary Fig. 6 Morphology and components of S_{Ni}. (a) Schematic illustration of the architecture of S_{Ni}. The dark grey core, the light grey mesoporous outermost layer, and the red spheres represent the dense silica, the mesoporous silica, and Ni, respectively. **(b)** TEM image of S_{Ni}. Scale bar, 50 nm. **(c)** HAADF-STEM image of S_{Ni}. Scale bar, 50 nm. **(d–f)** EDS mappings of S_{Ni}. Scale bars, 50 nm.

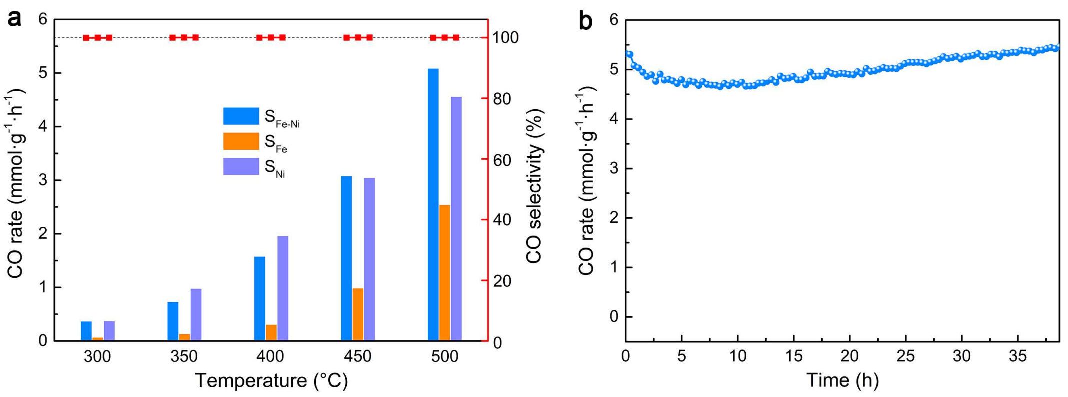

Supplementary Fig. 7 Thermocatalytic performance of CO_2 hydrogenation. (a) CO rate of different samples under various temperatures. **(b)** CO rate of $S_{Fe\text{-}Ni}$ under 500 ℃ during 38.7-hour testing. The red data points in (a) correspond to the selectivity towards production of CO.

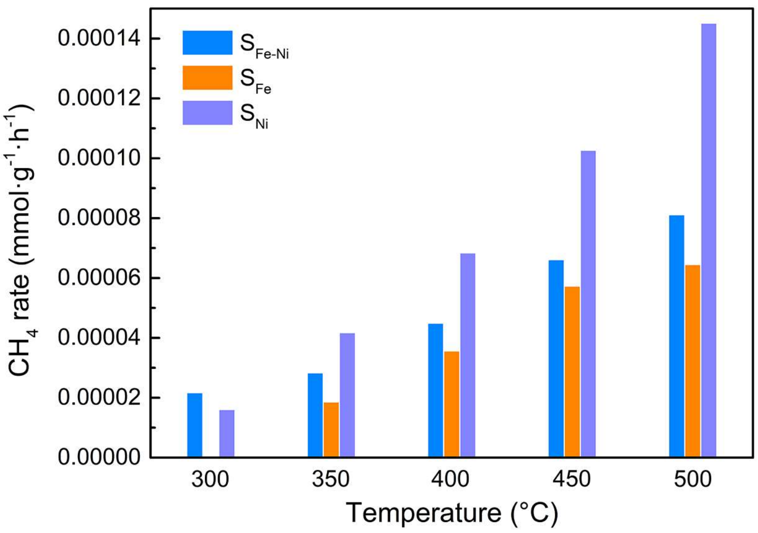

Supplementary Fig. 8 High selectivity towards CO. CH_4 production rates of the different samples under various temperatures are shown to be very low.

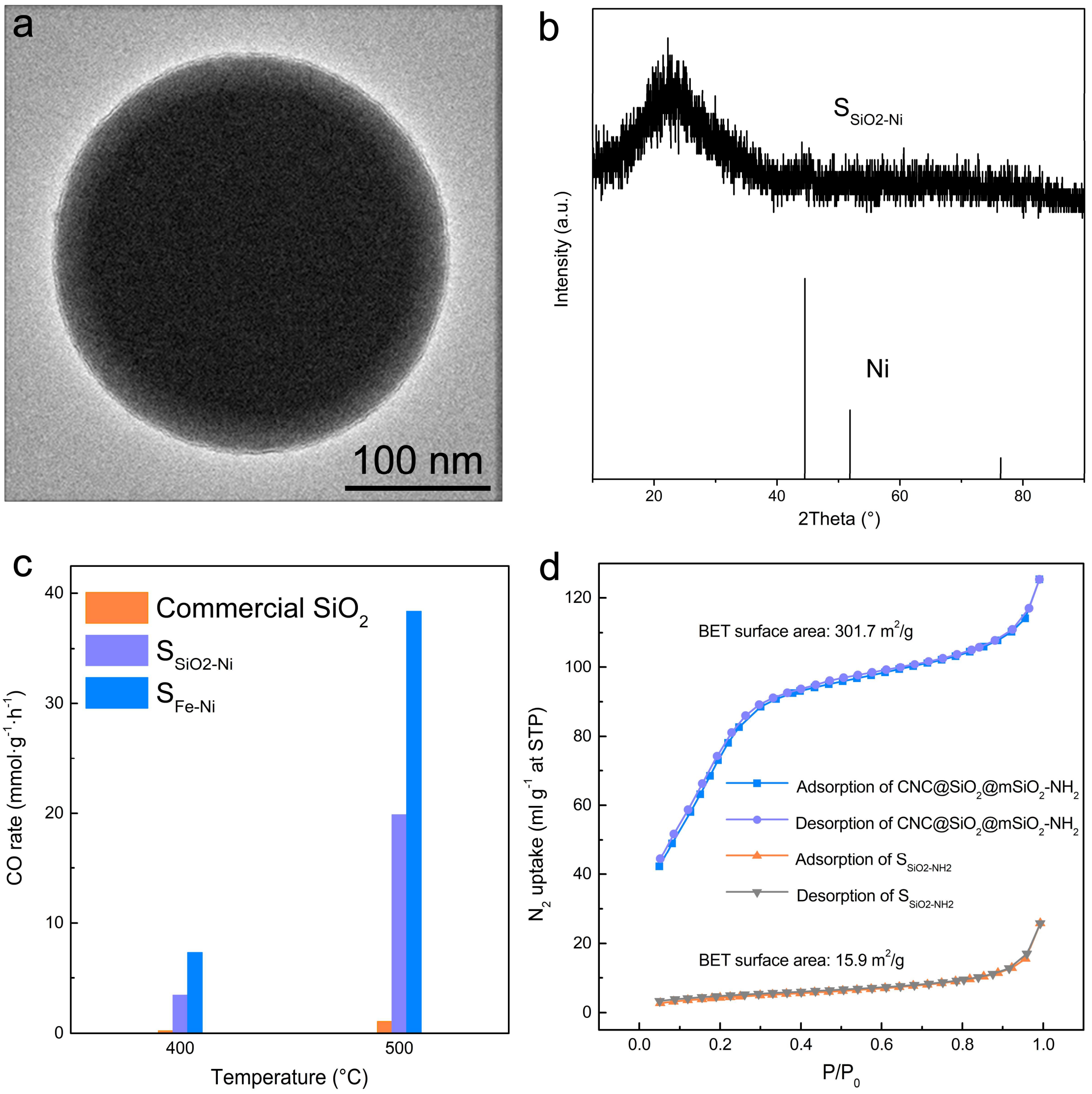

Supplementary Fig. 9 Function of mesopores in S_{Fe-Ni}. (a) TEM image of $S_{SiO2-Ni}$. Scale bar, 100 nm. **(b)** XRD patterns of $S_{SiO2-Ni}$ and the standard Ni (JCPDS 04-0850). **(c)** CO rates of $S_{SiO2-Ni}$ (testing condition: 6 mg of $S_{SiO2-Ni}$ was diluted by 24 mg of commercial SiO_2, $CO_2/H_2/N_2$ = 5/5/20 mL/min, ambient pressure), commercial SiO_2 (testing condition: 30 mg of commercial SiO_2, $CO_2/H_2/N_2$ = 5/5/20 mL/min, ambient pressure) and S_{Fe-Ni} (testing condition: 6 mg of S_{Fe-Ni} was diluted by 24 mg of commercial SiO_2, $CO_2/H_2/N_2$ = 5/5/20 mL/min, ambient pressure). **(d)** N_2 adsorption–desorption isotherms of CNC@SiO_2@mSiO_2-NH_2 and amino grafted S_{SiO2} ($S_{SiO2-NH2}$). Notably, the CO production rate was greatly improved to be ~ 40 mmol g_{cat}^{-1} h^{-1} by changing the space velocity from 20000 to 300000 mL g_{cat}^{-1} h^{-1}.

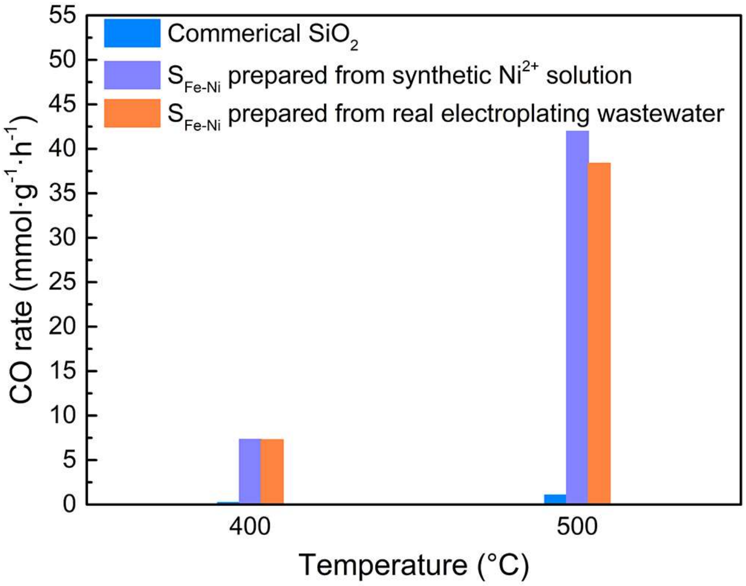

Supplementary Fig. 10 Effect of pollutants in wastewater on catalytic performance. CO rates of different S_{Fe-Ni} samples (testing condition: 6 mg of S_{Fe-Ni} was diluted by 24 mg of commercial SiO_2, $CO_2/H_2/N_2$ = 5/5/20 mL/min, ambient pressure) and commercial SiO_2 (testing condition: 30 mg of commercial SiO_2, $CO_2/H_2/N_2$ = 5/5/20 mL/min, ambient pressure). Notably, the CO production rate was greatly improved to be ~ 40 mmol g_{cat}^{-1} h^{-1} by changing the space velocity from 20000 to 300000 mL g_{cat}^{-1} h^{-1}.

The slightly lower CO rate of S_{Fe-Ni} prepared from real electroplating wastewater rather than S_{Fe-Ni} prepared from the synthetic Ni^{2+} solution might be ascribed to the slightly higher Fe loading for the latter (17.9 wt%) than the former (15.9 wt%) determined by ICP-OES. As **Fig. S7** shows, the Fe component exhibits a significant contribution to the production rate at 500 °C in thermocatalytic tests. While at 400 °C, this contribution is much smaller. That might be the reason for the same CO rate of these two kinds of S_{Fe-Ni} samples at 400 °C.

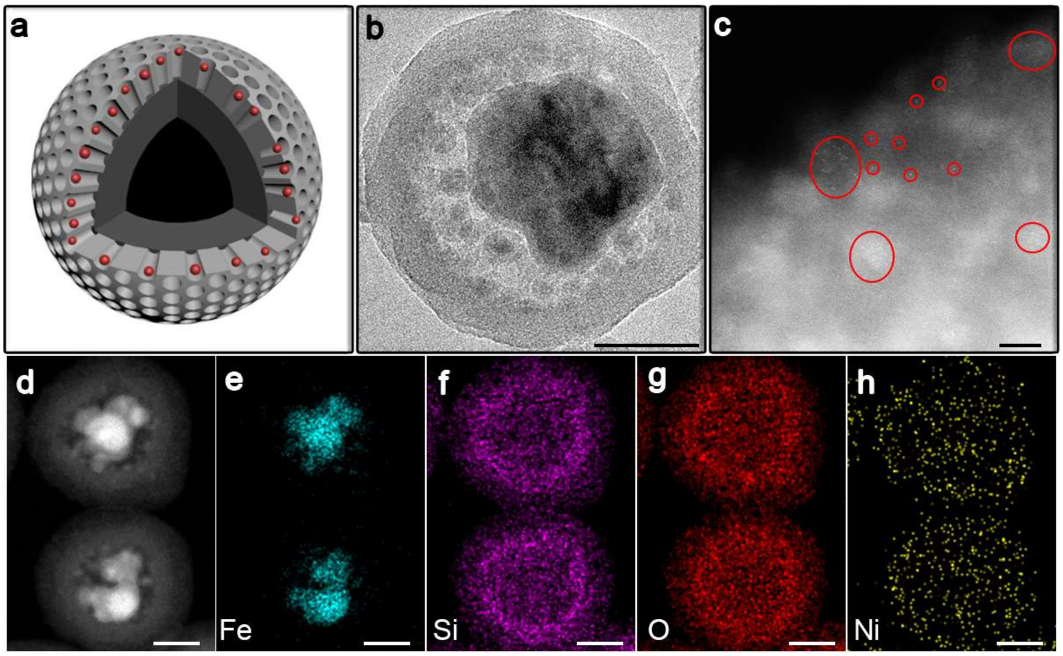

Supplementary Fig. 11 Morphology and components of $S_{Fe\text{-}Ni}$ (after). (a) Schematic illustration of the architecture of $S_{Fe\text{-}Ni}$ (after). The black core, the dark grey middle layer, the light grey mesoporous outermost layer, and the red spheres represent the Fe core after reduction, the dense silica, the mesoporous silica, and Ni, respectively. **(b)** TEM image of $S_{Fe\text{-}Ni}$ (after). Scale bar, 50 nm. **(c)** HAADF-STEM image of $S_{Fe\text{-}Ni}$ (after). Scale bar, 2 nm. **(d–h)** EDS mappings of $S_{Fe\text{-}Ni}$ (after). Scale bars, 50 nm.

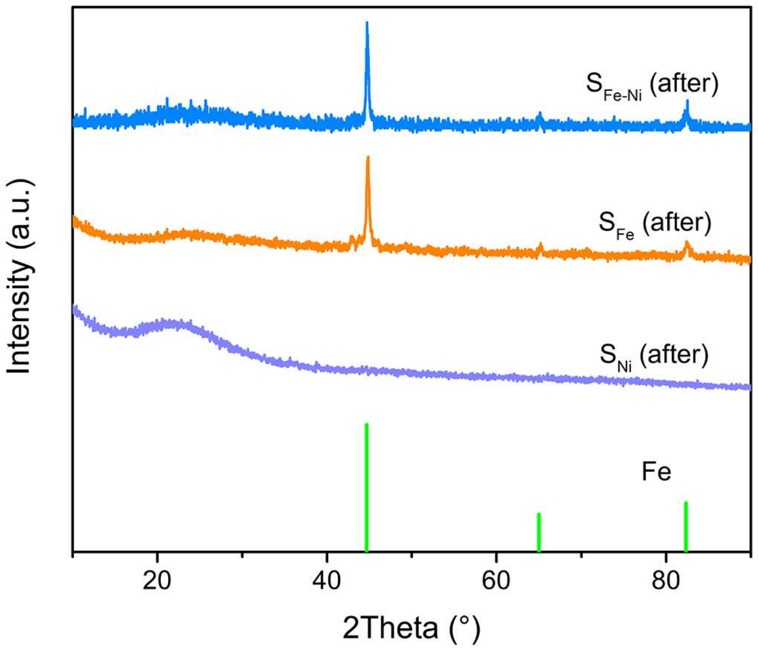

Supplementary Fig. 12 XRD patterns of the different samples and the standard Fe (JCPDS 06-0696). The used S_{Fe-Ni}, S_{Fe}, and S_{Ni} were denoted as S_{Fe-Ni} (after), S_{Fe} (after), and S_{Ni} (after), respectively.

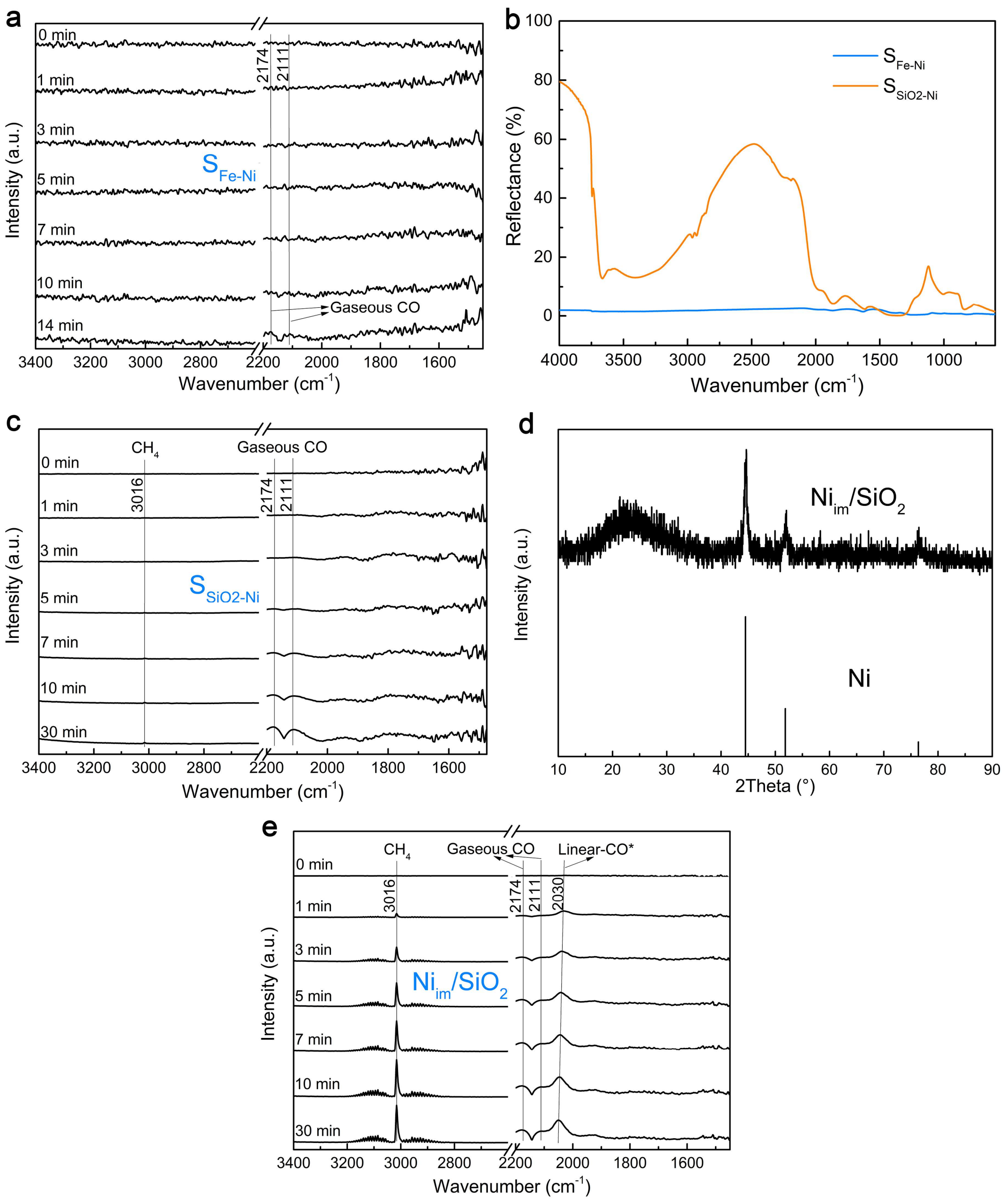

Supplementary Fig. 13 Study on reaction pathway. (a) In-situ DRIFTS spectra of $S_{Fe\text{-}Ni}$. **(b)** Diffuse reflectance spectra of $S_{Fe\text{-}Ni}$ and $S_{SiO2\text{-}Ni}$ from 600-4000 cm^{-1}. KBr was used as the referenced background. **(c)** In-situ DRIFTS spectra of $S_{SiO2\text{-}Ni}$. **(d)** XRD pattern of solid SiO_2 supported Ni nanoparticles (Ni_{im}/SiO_2) and the standard Ni (JCPDS 04-0850). **(e)** In-situ DRIFTS spectra of Ni_{im}/SiO_2. Test conditions of all samples in the in-situ DRIFTS experiments: ~20 mg of catalyst, CO_2/H_2 = 2.5/2.5 mL/min, ambient pressure.

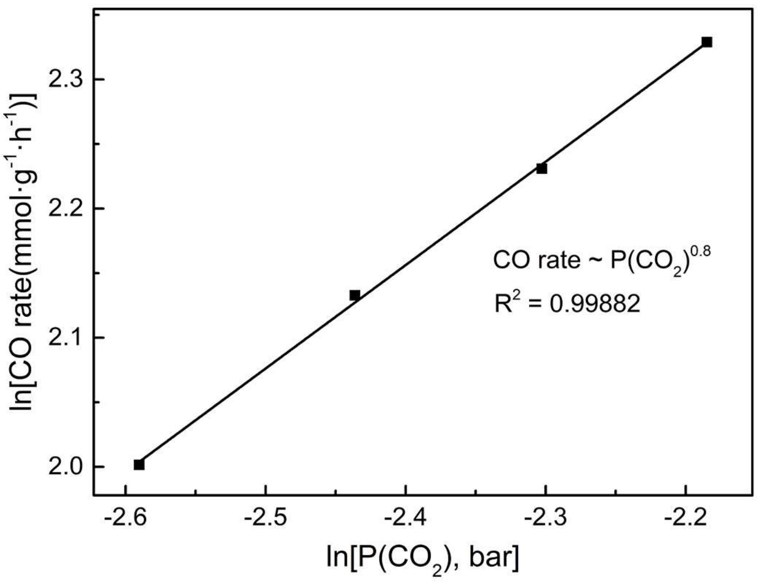

Supplementary Fig. 14 CO_2 rate order for $S_{Fe\text{-}Ni}$. CO_2 concentration was varied between 7.5% and 11.25% while H_2 concentration remained at 75% with conversions below 5%.

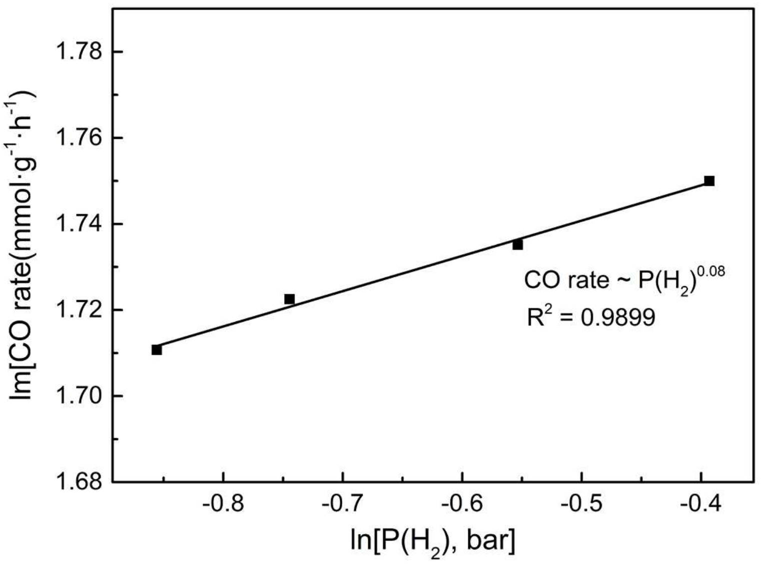

Supplementary Fig. 15 H_2 rate order for $S_{Fe\text{-}Ni}$. H_2 concentration was varied between 42.5% and 67.5% while CO_2 concentration remained at 12.5% with conversions below 5%.

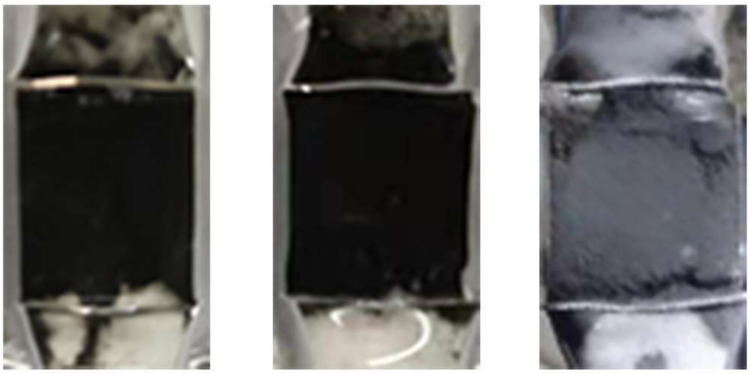

Supplementary Fig. 16 Enhanced light absorption by the Fe core. Photographs of the different fresh samples.

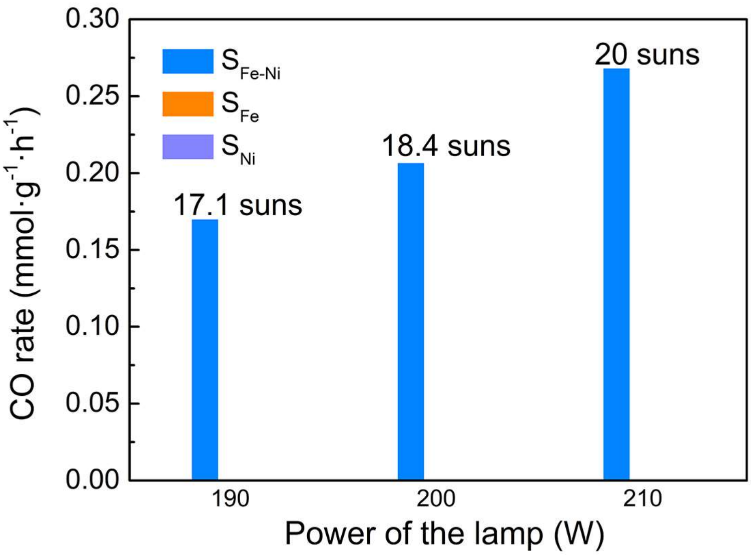

Supplementary Fig. 17 Enhanced photothermal catalytic performance of S_{Fe-Ni}. CO production rates of the different samples illuminated with a Xe arc lamp.

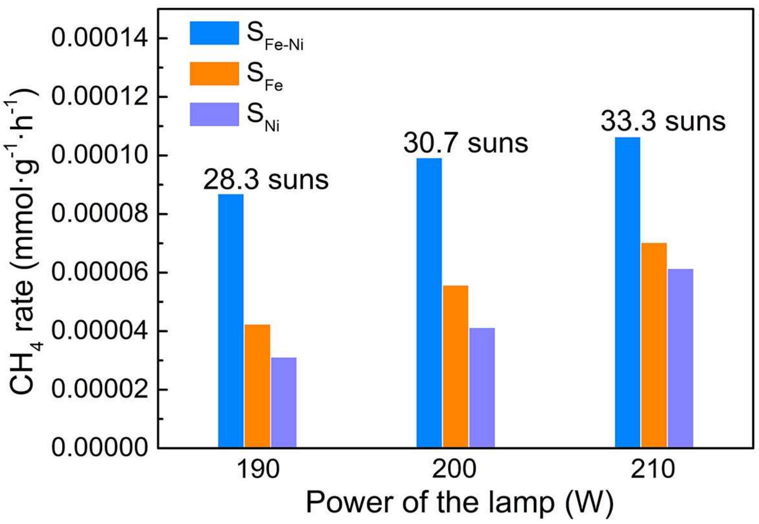

Supplementary Fig. 18 CH_4 production rates of the different samples illuminated with a Xe arc lamp supplemented by a light concentrator. The power of the lamp was set to be 190, 200, and 210 W.

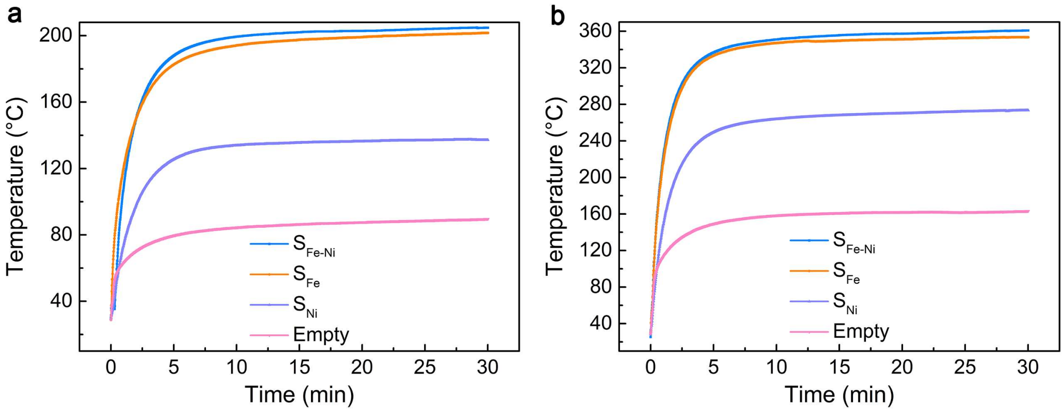

Supplementary Fig. 19 Surface temperature profiles of the different samples. The samples were illuminated with **(a)** a Xe arc lamp, of which the power was set to be 190 W, and **(b)** concentrated light from a Xe arc lamp, of which the power was set to be 190 W.

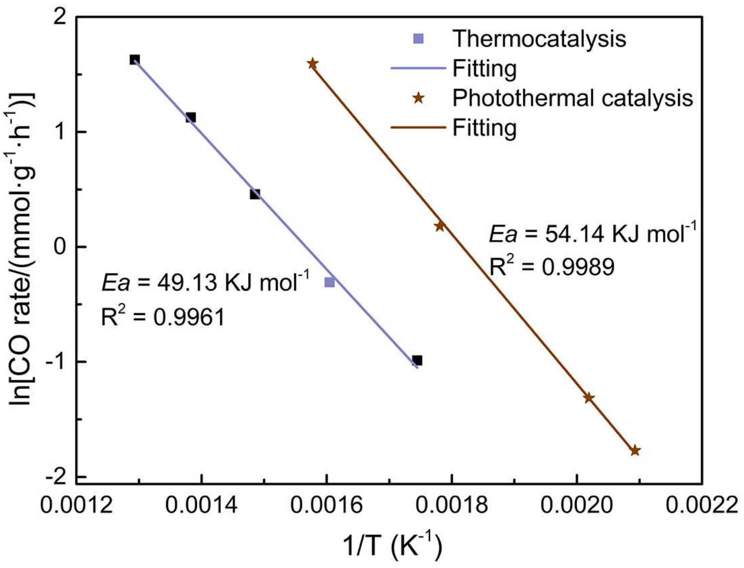

Supplementary Fig. 20 Arrhenius plot fitting from the CO rates in the thermal and light-driven processes. The temperatures in the light-driven process could be obtained from the source data file.

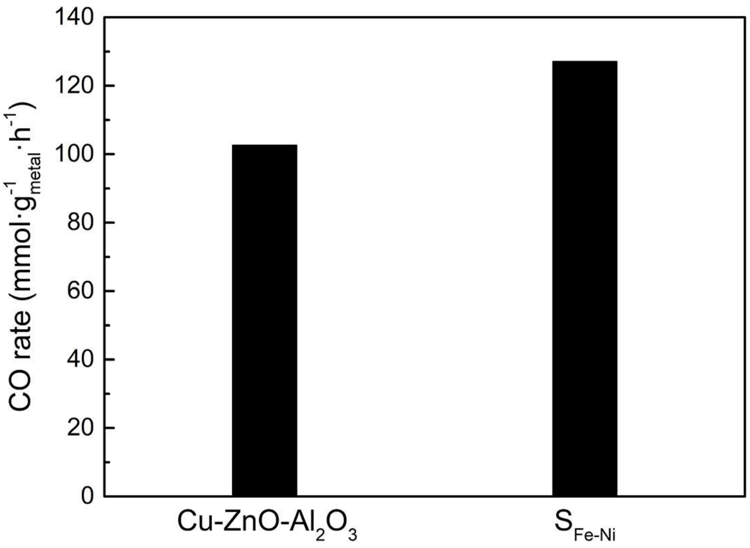

Supplementary Fig. 21 Enhanced photothermal catalytic performance. Photothermal catalytic CO production rates of Cu-ZnO-Al_2O_3 (testing condition: 1.5 mg of commercial Cu-ZnO-Al_2O_3 was diluted by 28.5 mg of commercial SiO_2, $CO_2/H_2/N_2$ = 2.5/2.5/5 mL/min, ambient pressure) and S_{Fe-Ni} (testing condition: 30 mg, $CO_2/H_2/N_2$ = 2.5/2.5/5 mL/min, ambient pressure). The Cu weight ratio of commercial Cu-ZnO-Al_2O_3 was determined by ICP-OES to be 42.9%. The diluted Cu-ZnO-Al_2O_3 sample (2.1 wt% Cu) in the testing condition has similar weight ratio of active metal as S_{Fe-Ni} (2.0 wt% Ni). The samples were illuminated with a Xe arc lamp supplemented with a light concentrator in the flow reactor. The power of the lamp was set to be 180 W, corresponding to the light intensity of 26.1 suns. The CO rates with the unit of 'mmol·g_{metal}^{-1}·h^{-1}' for S_{Fe-Ni} was calculated based on the mass of Ni, and the activity of the isolated Fe component was deducted by subtracting the CO rate of S_{Fe} under the same testing condition.

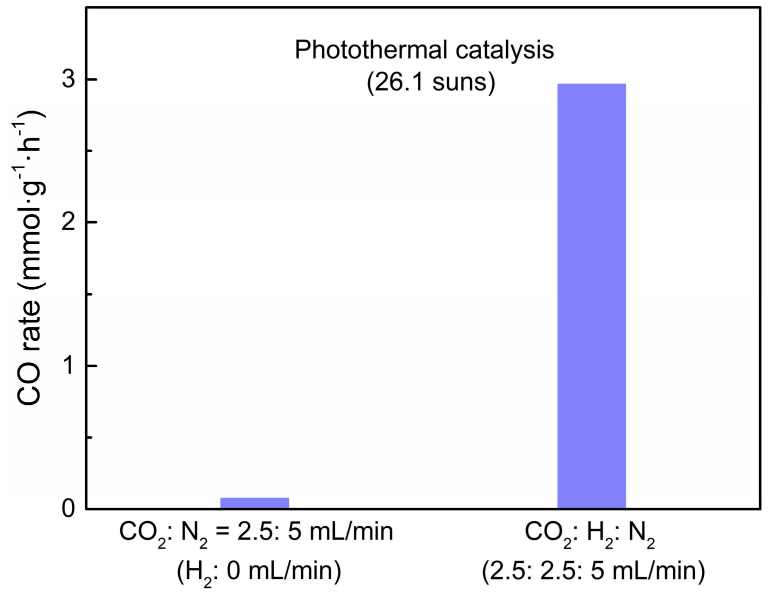

Supplementary Fig. 22 Evidence of reverse water-gas shift reaction. Catalytic performance of $S_{Fe\text{-}Ni}$ with and without H_2.

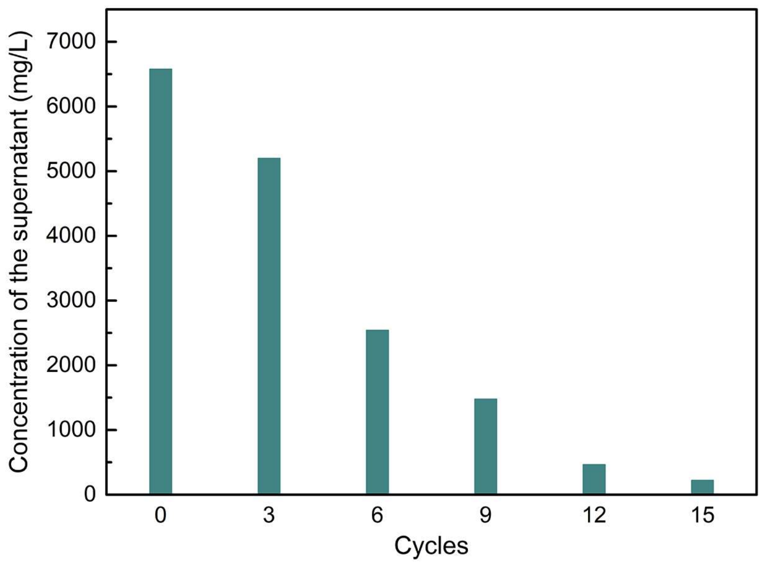

Supplementary Fig. 23 Cycle performance for removal of Ni. The concentration of the remaining Ni in the wastewater after numerous typical cycles.

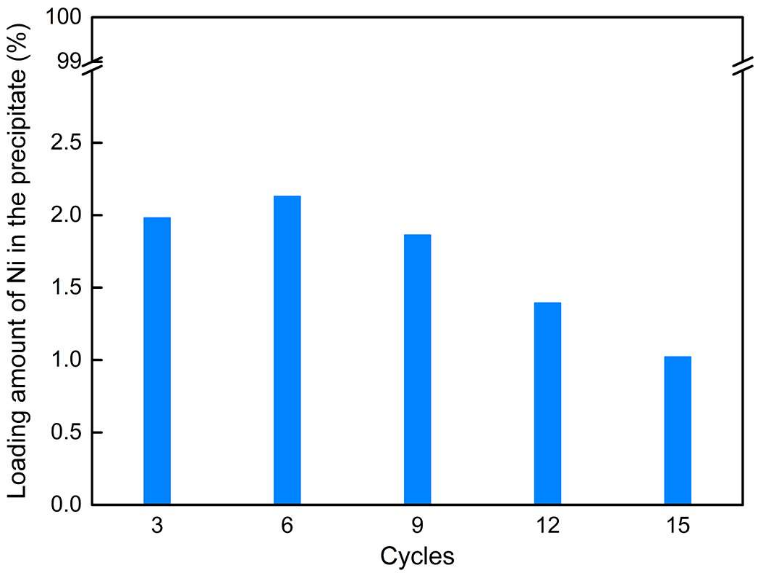

Supplementary Fig. 24 Cycle performance for loading Ni. The loading amount of Ni for $S_{Fe\text{-}Ni}$n after multiple typical cycles.

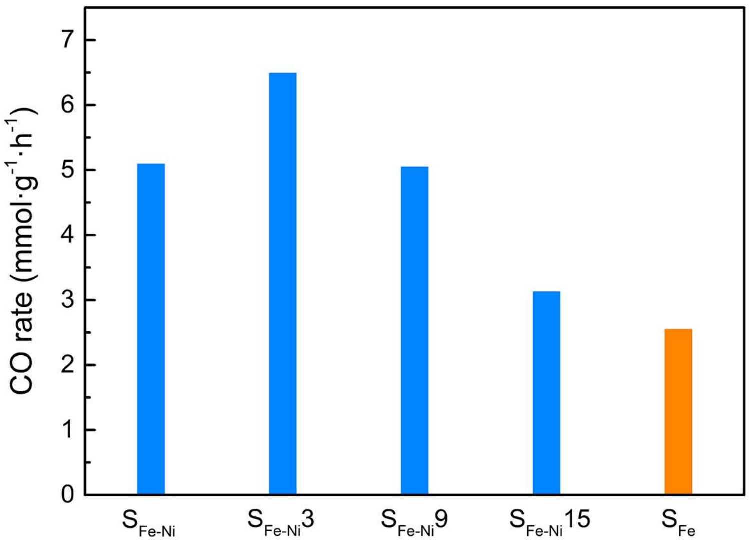

Supplementary Fig. 25 Cycle performance for CO_2 hydrogenation. Thermocatalytic performance of three typical $S_{Fe\text{-}Ni}n$ (n = 3, 9, and 15) samples at 500 °C.

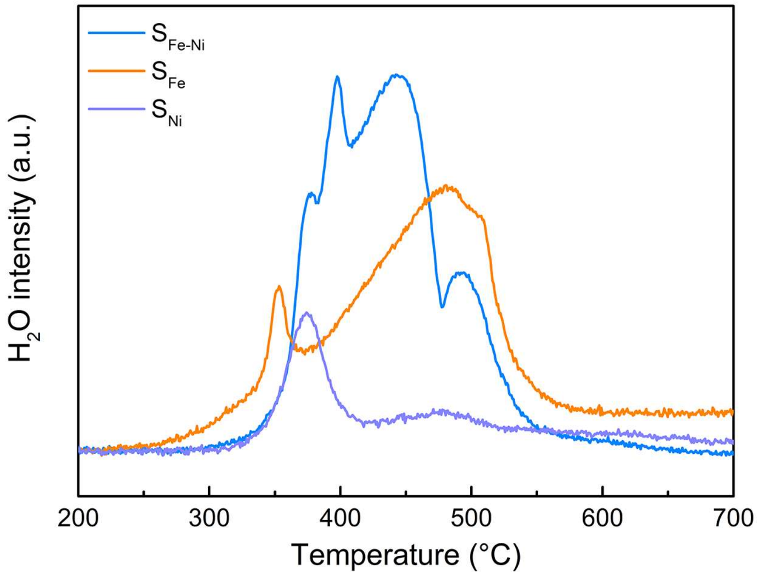

Supplementary Fig. 26 Evidence of complete reduction of metals. Temperature-programmed reduction (TPR) profiles of the samples.

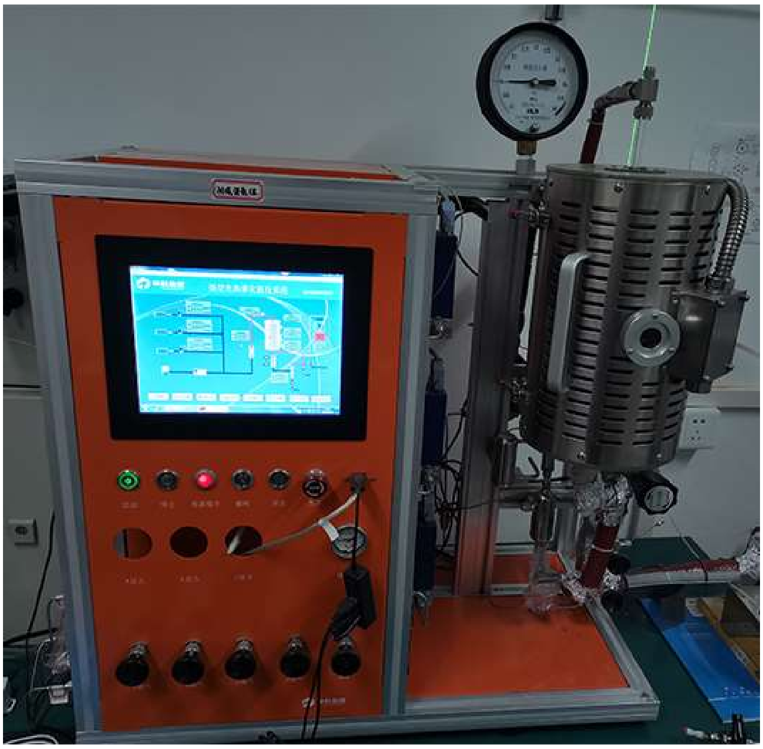

Supplementary Fig. 27 Reactor architecture. The photothermal reactor used in this work, which can operate in both thermal and photo modes with temperature readouts.

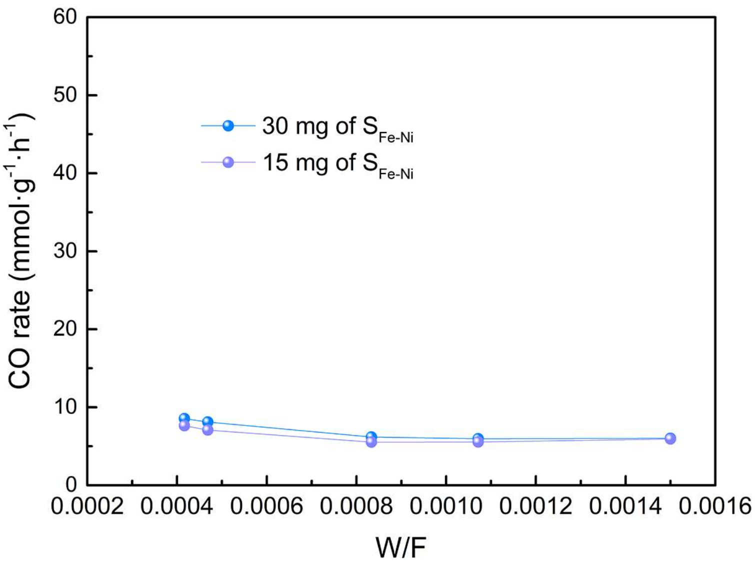

Supplementary Fig. 28 Space velocities dependent performances of $S_{Fe\text{-}Ni}$. Testing conditions: 15 or 30 mg of catalyst, T= 500 °C, $CO_2/H_2/N_2$ = 1: 1: 2. When the mass of the catalyst was 15 mg (denoted as $S_{Fe\text{-}Ni}$-15mg), the total flow rate of the inlet gas varied from 10 to 36 mL/min; When the mass of the catalyst was 30 mg (denoted as $S_{Fe\text{-}Ni}$-30mg) , the total flow rate of the inlet gas varied from 20 to 72 mL/min. *W* and *F* in the heading of the Abscissa represent mass of the catalyst (g) and total flow rate (mL/min), respectively.

The CO production rate for $S_{Fe\text{-}Ni}$-15mg approaches that for $S_{Fe\text{-}Ni}$-30mg under the same W/F value (0.00042-0.0015). Therefore, the influence of the external diffusion was eliminated under these testing conditions.

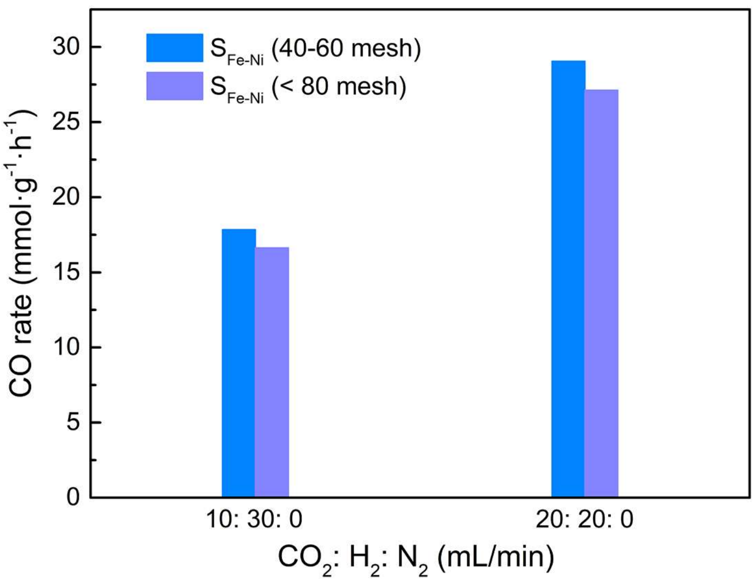

Supplementary Fig. 29 Thermocatalytic performances of $S_{Fe\text{-}Ni}$ with different particle sizes.

Testing conditions: 30 mg of catalyst, T= 500 °C.

The CO production rate for $S_{Fe\text{-}Ni}$ (40-60 mesh) approaches that for $S_{Fe\text{-}Ni}$ (< 80 mesh) under the same condition. Therefore, the thermocatalytic performance would not be dependent on the particle size (< 40 mesh). Combined with the calculations in the supplementary notes, the influence of the internal diffusion was eliminated under these testing conditions.

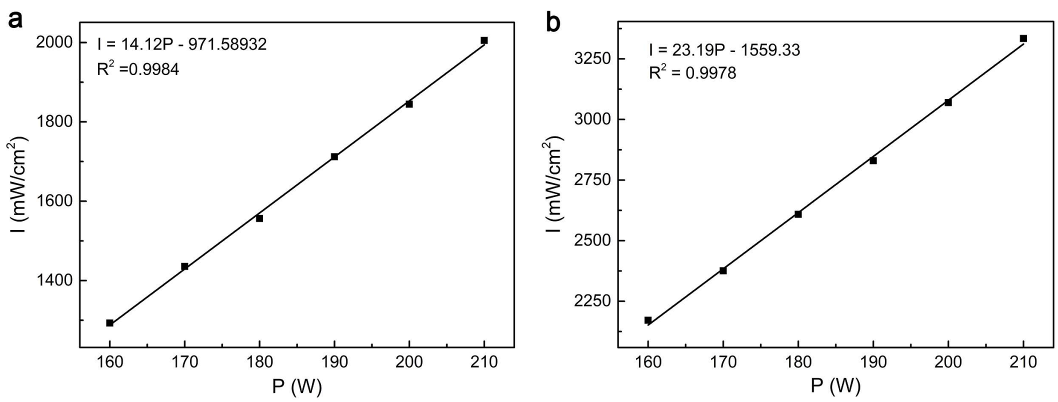

Supplementary Fig. 30 The relationship between the power of the lamp (P) and the light intensity (I). (a) without a concentrator, **(b)** with a concentrator.

Supplementary Table 1 Structure parameters extracted from the EXAFS fitting of Ni K-edge.

Sample	Shell	CN	R(Å)	σ^2(Å^2)	$\triangle E_0$ (eV)	R-factor
**NiO**	Ni-O	6	2.1	0.008	−3.381	0.002
	Ni-Ni	12	2.97	0.008	−3.381	
**Ni foil**	Ni-Ni	12	2.48	0.006	6.049	0.002
**S_{Fe-Ni}**	Ni-O	3.78±0.64	2.04±0.04	0.006	−2.879	0.003
	Ni-Ni	4.75±2.01	2.52±0.14	0.018	−1.006	
**S_{Fe-Ni} (after)**	Ni-O	3.75±0.39	2.1±0.02	0.008	9.201	0.012
	Ni-O-Ni	6.88±1.28	2.95±0.05	0.018	−7.347	

Supplementary Table 2 Thermocatalytic performance of Ni/SiO_2·Al_2O_3 and Ni_{im}/SiO_2.

Catalyst	m (mg)[a]	T (°C)[b]	P (Pa)[c]	CO selectivity (%)
Ni/SiO_2·Al_2O_3	50	500	101325	41.4
Ni_{im}/SiO_2	30	500	101325	55.8

[a]Testing conditions: CO_2:H_2:N_2 = 2.5:2.5:5 mL min^{-1}. [b]reaction temperature. [c]reaction pressure.

Supplementary Table 3 The concentration of different elements in the electroplating wastewater.

Elements	Ni	Na	Ca	K	Mn	Mg	Cu	Cr	Al	B	Si	P
**Concentration ($mg\ L^{-1}$)**	6576	70.99	10.22	3.87	0.93	0.73	0.08	0.20	0.59	320	0.52	0.67

The concentrations of Na, Ca, K, Mn, Mg, Cu and Cr in the wastewater were determined by the ICE3500 atomic absorption spectrophotometer (Thermo Fisher). The concentrations of Al, B, Si, and P in the wastewater were determined by the inductively coupled plasma atomic emission spectrometer (Prodigy, LEEMAN). The concentrations of Ni in the wastewater was determined by an Inductively coupled plasma source mass spectrometer (ICP-MS) (Aurora M90, Jenoptik).

Supplementary Table 4 The loading amount of Ni and Fe for the different samples.

Sample	Weight ratio of Ni	Weight ratio of Fe
**S_{Fe-Ni} (batch #1) from real electroplating waste water[a]**	2%	17%
**S_{Fe-Ni} (batch #2) from real electroplating waste water[a]**	1.9%	16.8%
**S_{Fe-Ni} (batch #3) from real electroplating waste water[a]**	2.1%	15.9%
**S_{Fe-Ni} from synthetic Ni^{2+} solution**	1.7%	17.9%
**S_{Fe}**	0	23%
**S_{Ni}**	2.6%	0

[a]Three batches of samples were prepared from the same wastewater to verify the reproducibility of the upcycling procedure.

Supplementary Table 5 The loading amount of B, Na and Ca (which were with the highest concentrations in the original electroplating wastewater) for different $S_{Fe\text{-}Ni}$ samples.

Sample	Weight ratio of B	Weight ratio of Na	Weight ratio of Ca
**$S_{Fe\text{-}Ni}$ (batch #1) from real electroplating wastewater**	0.23%	0.038%	0.14%
**$S_{Fe\text{-}Ni}$ (batch #2) from real electroplating wastewater**	0.24%	0.036%	0.097%
**$S_{Fe\text{-}Ni}$ (batch #3) from real electroplating wastewater**	0.24%	0.038%	0.13%
**$S_{Fe\text{-}Ni}$ from synthetic Ni^{2+} solution**	0	0.034%	0.21%

Three batches of $S_{Fe\text{-}Ni}$ samples from the same real electroplating wastewater were prepared to verify the reproducibility of the upcycling procedure.

The almost same loading amounts of B, Na and Ca in the three batches of the $S_{Fe\text{-}Ni}$ samples from real electroplating wastewater demonstrate the reproducibility of the upcycling procedure. Moreover, the $S_{Fe\text{-}Ni}$ sample prepared from manmade Ni^{2+} solution contains no B elements, again demonstrating that our catalysts were prepared from real electroplating wastewater. It is noticed that both the $S_{Fe\text{-}Ni}$ samples from wastewater and manmade Ni^{2+} solution contain negligible amounts of Na and Ca, which might be attributed to the existence of trace amounts of Na and Ca in the reagents or acid solution used to dissolve the samples.

Supplementary Table 6 Selectivity of CO for S_{Fe-Ni} tested under different conditions.

Testing condition	Temperature or power of lamp	CO selectivity (%)
Thermocatalysis	300 °C	99.9942
	350 °C	99.9961
	400 °C	99.9972
	450 °C	99.9979
	500 °C	99.9984
Photothermal catalysis	190 W	100
	200 W	100
	210 W	100
	190 W+[a]	99.9982
	200 W+[a]	99.9985
	210 W+[a]	99.9989

[a]The suffix '+' represents the illumination with the assist of a concentrator.

Supplementary Table 7 Comparison of the catalytic performance of our catalyst and the other reported photothermal catalysts tested in batch reactors.

Catalyst	Metal [wt%]	CO_2: H_2	Light source	Light intensity (mW/cm^2)	CO selec.	R_{max} ($mol\ g_{metal}^{-1}\ h^{-1}$)
Ni/Al_2O_3[4]	2.1	1:4	300 W Xe light (UV-Vis-NIR)	N/A	0.95%	2.3
Co/Al_2O_3[4]	2.5	1:4	300 W Xe light (UV-Vis-NIR)	N/A	0.49%	0.9
Pd/Al_2O_3[4]	2	1:4.1	300 W Xe light (UV-Vis-NIR)	N/A	1.36%	0.53
Pt/Al_2O_3[4]	2.4	1:4.1	300 W Xe light (UV-Vis-NIR)	N/A	84.85%	0.47
Ir/Al_2O_3[4]	2.8	1:4.1	300 W Xe light (UV-Vis-NIR)	N/A	36.74%	0.05
$Ni/N_{5.0}$-CeO_2[5]	10	1:1	300 W Xe light (UV-Vis-NIR)	2110	~100%	0.209
Ru/TiNT[6]	11.2	1:4	Newport solar simulator	150 + 210°C	N/A	0.1107
Co@CoN&C-1[7]	76.3	1:1	300 W Xe light	N/A	91.1%	0.17
Ni/80Ce-20Ti_SG[8]	10	1:4	300 W Xe light	N/A	N/A	0.17
$Pd@Nb_2O_5$[9]	0.5	1:1	300 W Xe light	2500	N/A	0.98
$S_{Fe\text{-}Ni}$ (this work)	Ni: 2	1:1	300 W Xe light (UV-Vis-NIR)	2800	> 99.8%	1.9*

*The CO rates were calculated based on the mass of Ni, and the activity of the isolated Fe component was deducted by subtracting the CO rate of S_{Fe} under the same testing condition.

Supplementary Table 8 The prices of nickel from different resources in Chinese Yuan (¥).

Source of Ni	Price
Ni from electroplating wastewater	−0.012 CNY g^{-1} to −0.018 CNY g^{-1}[a] (−0.0019 USD g^{-1} to −0.0028 USD g^{-1})
Ni from $Ni(NO_3)_2 \cdot 6H_2O$	64.46 CNY g^{-1}[b] (9.84 USD g^{-1})
Ni from $NiCl_2 \cdot 6H_2O$	11.1 CNY g^{-1}[c] (1.7 USD g^{-1})
Ni from $NiSO_4 \cdot 6H_2O$	15.14 CNY g^{-1}[d] (2.31 USD g^{-1})

[a]The price was calculated based on the charge of waste electroplating water treatment (¥80 m^{-3} – ¥120 m^{-3}) and the concentration of Ni in the wastewater (6.576 g L^{-1}). The negative sign means contrary to purchasing from the industry, the electroplating industry pays who acquires this Ni resource. [b]The price was calculated based on the price of $Ni(NO_3)_2 \cdot 6H_2O$ from Sigma-Aldrich (6504.9 CNY/500g) as of when this work was submitted. [c]The price was calculated based on the price of $NiCl_2 \cdot 6H_2O$ from Sigma-Aldrich (1372.16 CNY/500g) as of when this work was submitted. [d]The price was calculated based on the price of $NiSO_4 \cdot 6H_2O$ from Sigma-Aldrich (1689.19 CNY/500g) as of when this work was submitted.

Supplementary references

1. Dong, X., Jin, B., Kong, Z. & Dong, L. Intrinsic kinetics study of biogas methanation coupling with water gas shift over re-promoted Ni bifunctional catalysts. *Catalysts* **9** (2019).
2. Bui, P. P., Oyama, S. T., Takagaki, A., Carrow, B. P. & Nozaki, K. Reactions of 2-methyltetrahydropyran on silica-supported nickel phosphide in comparison with 2-methyltetrahydrofuran. *ACS Catal.* **6**, 4549-4558 (2016).
3. Fuller, E. N., Schettler, P. D. & Giddings, J. C. New Method for prediction of binary gas-phase diffusion Coefficients. *Ind. Eng. Chem.* **58**, 18-27 (1966).
4. Meng, X. *et al.* Photothermal conversion of CO_2 into CH_4 with H_2 over Group VIII nanocatalysts: an alternative approach for solar fuel production. *Angew. Chem. Int. Ed.* **53**, 11478-11482 (2014).
5. Jia, Z. *et al.* Selective photothermal reduction of CO_2 to CO over Ni-nanoparticle/N-doped CeO_2 nanocomposite catalysts. *ACS Appl. Nano Mater.* **4**, 10485-10494 (2021).
6. Novoa-Cid, M. & Baldovi, H. G. Study of the photothermal catalytic mechanism of CO_2 reduction to CH_4 by ruthenium nanoparticles supported on titanate nanotubes. *Nanomaterials* **10**, 2212 (2020).
7. Ning, S. *et al.* Microstructure Induced Thermodynamic and Kinetic Modulation to Enhance CO2 Photothermal Reduction: A Case of Atomic-Scale Dispersed Co–N Species Anchored Co@C Hybrid. *ACS Catal.* **10**, 4726-4736 (2020).
8. Kho, E. T., Jantarang, S., Zheng, Z., Scott, J. & Amal, R. Harnessing the

beneficial attributes of ceria and titania in a mixed-oxide support for nickel-catalyzed photothermal CO_2 methanation. *Engineering* **3**, 393-401 (2017).

9. Jia, J. *et al.* Visible and near-infrared photothermal catalyzed hydrogenation of gaseous CO_2 over nanostructured Pd@Nb_2O_5. *Adv. Sci.* **3**, 1600189 (2016).
